# Supplementary material for: HIVprotI: an integrated web based platform for prediction and design of HIV proteins inhibitors
Source: J Cheminform. 2018 Mar 9;10:12. doi: 10.1186/s13321-018-0266-y (PMC5845081; doi:10.1186/s13321-018-0266-y)
Supplement: Supplementary file 2 — Additional file 2. Source code of HIVProtI web server. [file 13321_2018_266_MOESM2_ESM.zip › HIVprotI_Source-code/hivprotI/weka-3-6-6/doc/weka/filters/unsupervised/attribute/MathExpression.html]

MathExpression


---


|  |  |  |
| --- | --- | --- |
| **Overview** \| **Package** \| **Class** \| **Tree** \| **Deprecated** \| **Index** \| **Help** \| **Weka's home** | |  |
| **PREV CLASS**   **NEXT CLASS** | **FRAMES**    **NO FRAMES**     **All Classes** |  |
| SUMMARY: NESTED \| FIELD \| CONSTR \| METHOD | DETAIL: FIELD \| CONSTR \| METHOD |  |


---


## weka.filters.unsupervised.attribute Class MathExpression

```
java.lang.Object
  weka.filters.Filter
      weka.filters.unsupervised.attribute.PotentialClassIgnorer
          weka.filters.unsupervised.attribute.MathExpression
```

**All Implemented Interfaces:**: java.io.Serializable, CapabilitiesHandler, OptionHandler, RevisionHandler, UnsupervisedFilter

---

``` public class MathExpression extends PotentialClassIgnorer implements UnsupervisedFilter ```

Modify numeric attributes according to a given expression


Valid options are:

```
 -unset-class-temporarily
  Unsets the class index temporarily before the filter is
  applied to the data.
  (default: no)
```

```
 -E <expression>
  Specify the expression to apply. Eg. pow(A,6)/(MEAN+MAX)
  Supported operators are +, -, *, /, pow, log,
  abs, cos, exp, sqrt, tan, sin, ceil, floor, rint, (, ), 
  MEAN, MAX, MIN, SD, COUNT, SUM, SUMSQUARED, ifelse
```

```
 -R <index1,index2-index4,...>
  Specify list of columns to ignore. First and last are valid
  indexes. (default none)
```

```
 -V
  Invert matching sense (i.e. only modify specified columns)
```

**Version:**
:   $Revision: 5543 $

**Author:**
:   Eibe Frank (eibe@cs.waikato.ac.nz), Prados Julien (julien.prados@cui.unige.ch)

**See Also:**: `MathematicalExpression`, Serialized Form

---

| **Field Summary** | |
| --- | --- |
| `static java.lang.String` | `m_defaultExpression`             The default modification expression |


**Constructor Summary**

`MathExpression()`
  
          Constructor


| **Method Summary** | |
| --- | --- |
| `boolean` | `batchFinished()`             Signify that this batch of input to the filter is finished. |
| `java.lang.String` | `expressionTipText()`             Returns the tip text for this property |
| `Capabilities` | `getCapabilities()`             Returns the Capabilities of this filter. |
| `java.lang.String` | `getExpression()`             Get the expression |
| `java.lang.String` | `getIgnoreRange()`             Get the current range selection. |
| `boolean` | `getInvertSelection()`             Get whether the supplied columns are to be select or unselect |
| `java.lang.String[]` | `getOptions()`             Gets the current settings of the filter. |
| `java.lang.String` | `getRevision()`             Returns the revision string. |
| `java.lang.String` | `globalInfo()`             Returns a string describing this filter |
| `java.lang.String` | `ignoreRangeTipText()`             Returns the tip text for this property |
| `boolean` | `input(Instance instance)`             Input an instance for filtering. |
| `java.lang.String` | `invertSelectionTipText()`             Returns the tip text for this property |
| `java.util.Enumeration` | `listOptions()`             Returns an enumeration describing the available options. |
| `static void` | `main(java.lang.String[] argv)`             Main method for testing this class. |
| `void` | `setExpression(java.lang.String expr)`             Set the expression to apply |
| `void` | `setIgnoreRange(java.lang.String rangeList)`             Set which attributes are to be ignored |
| `boolean` | `setInputFormat(Instances instanceInfo)`             Sets the format of the input instances. |
| `void` | `setInvertSelection(boolean invert)`             Set whether selected columns should be select or unselect. |
| `void` | `setOptions(java.lang.String[] options)`             Parses a given list of options. |

**Methods inherited from class weka.filters.unsupervised.attribute.PotentialClassIgnorer**

`getIgnoreClass, getOutputFormat, ignoreClassTipText, setIgnoreClass`

**Methods inherited from class weka.filters.Filter**

`batchFilterFile, filterFile, getCapabilities, isFirstBatchDone, isNewBatch, isOutputFormatDefined, makeCopies, makeCopy, numPendingOutput, output, outputPeek, toString, useFilter, wekaStaticWrapper`

**Methods inherited from class java.lang.Object**

`equals, getClass, hashCode, notify, notifyAll, wait, wait, wait`

**Field Detail**

### m\_defaultExpression

```
public static final java.lang.String m_defaultExpression
```

:   The default modification expression

    **See Also:**: Constant Field Values


**Constructor Detail**

### MathExpression

```
public MathExpression()
```

:   Constructor


**Method Detail**

### globalInfo

```
public java.lang.String globalInfo()
```

:   Returns a string describing this filter

    :   **Returns:**: a description of the filter suitable for displaying in the explorer/experimenter gui

---


### getCapabilities

```
public Capabilities getCapabilities()
```

:   Returns the Capabilities of this filter.

    :   **Specified by:**: `getCapabilities` in interface `CapabilitiesHandler` **Overrides:**: `getCapabilities` in class `Filter`
    :   **Returns:**: the capabilities of this object **See Also:**: `Capabilities`

---


### setInputFormat

```
public boolean setInputFormat(Instances instanceInfo)
                       throws java.lang.Exception
```

:   Sets the format of the input instances.

    :   **Overrides:**: `setInputFormat` in class `PotentialClassIgnorer`
    :   **Parameters:**: `instanceInfo` - an Instances object containing the input instance structure (any instances contained in the object are ignored - only the structure is required). **Returns:**: true if the outputFormat may be collected immediately **Throws:**: `java.lang.Exception` - if the input format can't be set successfully

---


### input

```
public boolean input(Instance instance)
              throws java.lang.Exception
```

:   Input an instance for filtering. Filter requires all
    training instances be read before producing output.

    :   **Overrides:**: `input` in class `Filter`
    :   **Parameters:**: `instance` - the input instance **Returns:**: true if the filtered instance may now be collected with output(). **Throws:**: `java.lang.IllegalStateException` - if no input format has been set.: `java.lang.NullPointerException` - if the input format has not been defined.: `java.lang.Exception` - if the input instance was not of the correct format or if there was a problem with the filtering.

---


### batchFinished

```
public boolean batchFinished()
                      throws java.lang.Exception
```

:   Signify that this batch of input to the filter is finished.
    If the filter requires all instances prior to filtering,
    output() may now be called to retrieve the filtered instances.

    :   **Overrides:**: `batchFinished` in class `Filter`
    :   **Returns:**: true if there are instances pending output **Throws:**: `java.lang.IllegalStateException` - if no input structure has been defined: `java.lang.NullPointerException` - if no input structure has been defined,: `java.lang.Exception` - if there was a problem finishing the batch.

---


### setOptions

```
public void setOptions(java.lang.String[] options)
                throws java.lang.Exception
```

:   Parses a given list of options.


    Valid options are:

    ```
     -unset-class-temporarily
      Unsets the class index temporarily before the filter is
      applied to the data.
      (default: no)
    ```

    ```
     -E <expression>
      Specify the expression to apply. Eg. pow(A,6)/(MEAN+MAX)
      Supported operators are +, -, *, /, pow, log,
      abs, cos, exp, sqrt, tan, sin, ceil, floor, rint, (, ), 
      MEAN, MAX, MIN, SD, COUNT, SUM, SUMSQUARED, ifelse
    ```

    ```
     -R <index1,index2-index4,...>
      Specify list of columns to ignore. First and last are valid
      indexes. (default none)
    ```

    ```
     -V
      Invert matching sense (i.e. only modify specified columns)
    ```

    :   **Specified by:**: `setOptions` in interface `OptionHandler` **Overrides:**: `setOptions` in class `PotentialClassIgnorer`
    :   **Parameters:**: `options` - the list of options as an array of strings **Throws:**: `java.lang.Exception` - if an option is not supported

---


### getOptions

```
public java.lang.String[] getOptions()
```

:   Gets the current settings of the filter.

    :   **Specified by:**: `getOptions` in interface `OptionHandler` **Overrides:**: `getOptions` in class `PotentialClassIgnorer`
    :   **Returns:**: an array of strings suitable for passing to setOptions

---


### listOptions

```
public java.util.Enumeration listOptions()
```

:   Returns an enumeration describing the available options.

    :   **Specified by:**: `listOptions` in interface `OptionHandler` **Overrides:**: `listOptions` in class `PotentialClassIgnorer`
    :   **Returns:**: an enumeration of all the available options.

---


### expressionTipText

```
public java.lang.String expressionTipText()
```

:   Returns the tip text for this property

    :   **Returns:**: tip text for this property suitable for displaying in the explorer/experimenter gui

---


### setExpression

```
public void setExpression(java.lang.String expr)
```

:   Set the expression to apply

    :   **Parameters:**: `expr` - a mathematical expression to apply

---


### getExpression

```
public java.lang.String getExpression()
```

:   Get the expression

    :   **Returns:**: the expression

---


### invertSelectionTipText

```
public java.lang.String invertSelectionTipText()
```

:   Returns the tip text for this property

    :   **Returns:**: tip text for this property suitable for displaying in the explorer/experimenter gui

---


### getInvertSelection

```
public boolean getInvertSelection()
```

:   Get whether the supplied columns are to be select or unselect

    :   **Returns:**: true if the supplied columns will be kept

---


### setInvertSelection

```
public void setInvertSelection(boolean invert)
```

:   Set whether selected columns should be select or unselect. If true the
    selected columns are modified. If false the selected columns are not
    modified.

    :   **Parameters:**: `invert` - the new invert setting

---


### ignoreRangeTipText

```
public java.lang.String ignoreRangeTipText()
```

:   Returns the tip text for this property

    :   **Returns:**: tip text for this property suitable for displaying in the explorer/experimenter gui

---


### getIgnoreRange

```
public java.lang.String getIgnoreRange()
```

:   Get the current range selection.

    :   **Returns:**: a string containing a comma separated list of ranges

---


### setIgnoreRange

```
public void setIgnoreRange(java.lang.String rangeList)
```

:   Set which attributes are to be ignored

    :   **Parameters:**: `rangeList` - a string representing the list of attributes. Since the string will typically come from a user, attributes are indexed from 1.

---


### getRevision

```
public java.lang.String getRevision()
```

:   Returns the revision string.

    :   **Specified by:**: `getRevision` in interface `RevisionHandler` **Overrides:**: `getRevision` in class `Filter`
    :   **Returns:**: the revision

---


### main

```
public static void main(java.lang.String[] argv)
```

:   Main method for testing this class.

    :   **Parameters:**: `argv` - should contain arguments to the filter: use -h for help


---


|  |  |  |
| --- | --- | --- |
| **Overview** \| **Package** \| **Class** \| **Tree** \| **Deprecated** \| **Index** \| **Help** \| **Weka's home** | |  |
| **PREV CLASS**   **NEXT CLASS** | **FRAMES**    **NO FRAMES**     **All Classes** |  |
| SUMMARY: NESTED \| FIELD \| CONSTR \| METHOD | DETAIL: FIELD \| CONSTR \| METHOD |  |


---
